# Navigation System–Assisted vs Freehand Cannulated Screw Fixation for Femoral Neck Fractures: Protocol for a Multicenter Randomized Controlled Trial

**DOI:** 10.2196/88180

**Published:** 2026-06-23

**Authors:** Meng Li, Dake Tong, Meng Cheng, Jiantao Li, Fang Ji, Wei Zhang

**Affiliations:** 1 Fourth Medical Center of PLA General Hospital Beijing, Beijing China; 2 Shanghai Ninth People’s Hospital Shanghai, Shanghai China

**Keywords:** randomized controlled trial, femoral neck fractures, navigation system, three cannulated screws, freehand technique, intraoperative fluoroscopy

## Abstract

**Background:**

The incidence of femoral neck fractures (FNFs) is increasing, primarily due to an aging population and an increased incidence of high-energy trauma. Although fixation with three cannulated screws (TCS) is the most commonly used surgical technique, it has limitations, including suboptimal operative accuracy and dependence on repeated intraoperative fluoroscopy. In response, advanced navigation systems have been developed to improve surgical precision and outcomes.

**Objective:**

This multicenter, prospective, randomized controlled superiority trial will compare surgical outcomes between an intelligent navigation system and the conventional freehand technique for FNF, as well as long-term fracture healing and complication rates.

**Methods:**

This multicenter, prospective, randomized controlled superiority trial will compare navigation system–assisted TCS fixation with conventional freehand TCS fixation in patients with FNF from June 2024 to October 2026. Eligible participants are adults aged ≥18 years diagnosed with FNF who are indicated for internal fixation. The primary outcome is the frequency of intraoperative fluoroscopy. Secondary outcomes include the neck-shaft angle, guide wire placement quality, operative time, intraoperative blood loss, fracture healing status, the Harris Hip Score, the 36-Item Short Form Health Survey, and the Zarit Caregiver Burden Score. The sample size calculation indicated that 150 (50%) participants per group (N=300) would be needed to detect a superiority margin of 15.4 fluoroscopy images with 80% power and a 1-sided α of .025, accounting for a 15% dropout rate. All patients will undergo 12 months of follow-up. Data analysis will compare groups using 1-sided *t* tests and Mann-Whitney *U* tests, supplemented by linear mixed-effects models with study site as a random effect.

**Results:**

The study was funded in May 2024 and received initial ethics approval from the institutional review board. Patient enrollment commenced in June 2024 and is projected to be completed by October 2026, with a target sample size of 300 participants. The final 12-month follow-up is expected to be completed by October 2027. As of manuscript submission, recruitment is ongoing, and no data analysis has been performed.

**Conclusions:**

Successful completion of this study may introduce a new strategy for the management of FNF and help establish a standardized protocol for the use of navigation systems, potentially enhancing clinical outcomes and reducing complication rates.

**Trial Registration:**

ClinicalTrials.gov NCT06713018; https://clinicaltrials.gov/study/NCT06713018

**International Registered Report Identifier (IRRID):**

DERR1-10.2196/88180

## Introduction

Hip fractures constitute a significant global health issue. They contribute greatly to morbidity, disability, and mortality, and they impose heavy demands on health care resources worldwide [1,2]. By 2050, the annual incidence of hip fractures is projected to rise to approximately 6.26 million. Almost half of these cases are expected to occur in Asia, particularly in China [3,4]. Each year, more than 1 million new femoral neck fractures (FNFs) are reported globally, with a rising incidence among younger populations [5]. The fixation of these fractures is inherently more technically complex. Despite advances in management, these fractures continue to be associated with a high complication rate. This can be explained by intrinsic factors, such as high-energy trauma and precarious vascularity, as well as iatrogenic contributors, including delays in diagnosis, imperfect fracture reduction, and insufficient fixation [6-8].

Reduction and internal fixation are the main treatments for FNF in young and middle-aged adults, typically using multiple cannulated screws or a dynamic hip screw [9,10]. However, a recent network meta-analysis of 33 randomized trials involving 5703 patients demonstrated that internal fixation carries substantially higher reoperation risks than arthroplasty (cannulated screws: odds ratio 9.98; dynamic hip screw: odds ratio 5.07) and yields inferior functional outcomes measured by the Harris Hip Score and the EQ-5D [11]. These findings underscore the importance of optimizing internal fixation techniques, particularly when femoral head preservation is desired. Most implants require precise guide wire positioning, traditionally achieved using a drill guide or alignment jig. In minimally invasive settings, achieving this precision without direct visualization is challenging, as poor positioning may cause malalignment and implant cutout [11-14].

A variety of advanced technologies, including orthopedic surgical robotic navigation, computer-assisted navigation systems, and intelligent minimally invasive visualization platforms, have been developed to enhance the precision and safety of cannulated screw placement and other implants procedures while reducing access morbidity and radiation exposure [12-14]. In the navigation system group, the first-pass success rate of TCS implantation reached 93.3%, compared to 60% in the freehand group, demonstrating a statistically significant difference (*χ*^²^=4.7; *P*=.03) [15]. According to a previous review, the primary success rate of other navigation systems ranged from approximately 93% to 99% in recent years, indicating a substantial improvement over freehand techniques [16-18]. Moreover, Schep et al [19] reported that a fluoroscopy-based navigation system achieved an accuracy of 1.17 mm, whereas Browbank et al [20] reported a precision of 3.4 mm for their X-ray–based mechanical guidance device. Therefore, a statistically significant and clinically meaningful reduction in fluoroscopy frequency would provide direct evidence of the navigation system’s superiority in terms of surgical efficiency and radiation safety [21].

Despite these innovations, current techniques, ranging from the conventional freehand approach to more technologically advanced solutions, still fall short of concurrently meeting the essential requirements of procedural simplicity, high accuracy, and minimal radiation exposure [22]. Some navigation systems have limitations, such as disruption of the conventional surgical workflow, increased operative time, and excessive preoperative imaging collection, which restrict their clinical application [23-25].

To address the aforementioned limitations, the StarNav-1 Orthopedic Surgical Navigation System was developed through an academic-industry collaboration between our research group and Visual MedTech Co, Ltd. This system effectively facilitates fracture reduction in long bone, periarticular, and spinal fractures. It is compatible with conventional internal implants, such as plates and intramedullary nails. It enables precise implantation with millimeter-level accuracy. It has obtained medical device registration in China. Preliminary cadaveric and sawbones validation studies have shown satisfactory outcomes. Preliminary unpublished data from the pilot phase demonstrated a reduction of approximately 15.2 fluoroscopy images per operation in the navigated group, with a first-pass guide wire success rate of 94.2%.

Building upon prior research, this multicenter, prospective, randomized controlled trial is designed to compare surgical outcomes between navigation system–assisted fixation and conventional freehand treatment for FNF, as well as the discrepancies in long-term fracture healing and complication rates. In this approach, the StarNav.1 Orthopedic Surgical Navigation System, provided by Visual MedTech Co, Ltd, will be used to guide implant positioning and fixation. The successful completion of this study will introduce a new strategy and establish a standardized protocol for the use of navigation systems in the management of these fractures.

## Methods

### Trial Design and Study Setting

**Figure 1:**
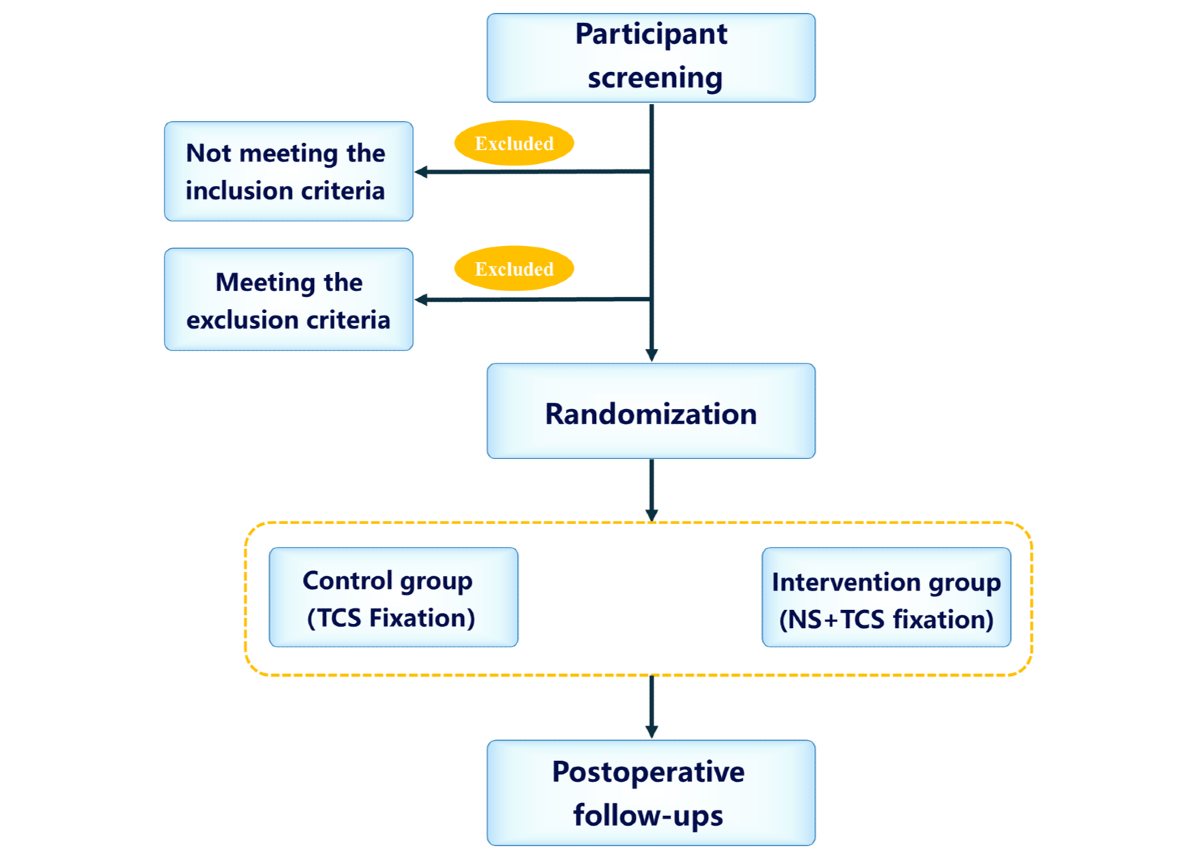
Overview of the participation in the clinical study. NS: navigation system; TCS: three cannulated screws.

This prospective, multicenter, randomized controlled clinical trial was designed to determine the superiority of the intervention. The study will take place at the Chinese People’s Liberation Army General Hospital and collaborating institutions from June 2024 to October 2026. Patients will be randomly allocated to 1 of 2 treatment groups (Figure 1): group 1—three cannulated screws (TCS) and group 2—navigation system–assisted TCS (NS+TCS).

### Description of the Novel Device

**Figure 2:**
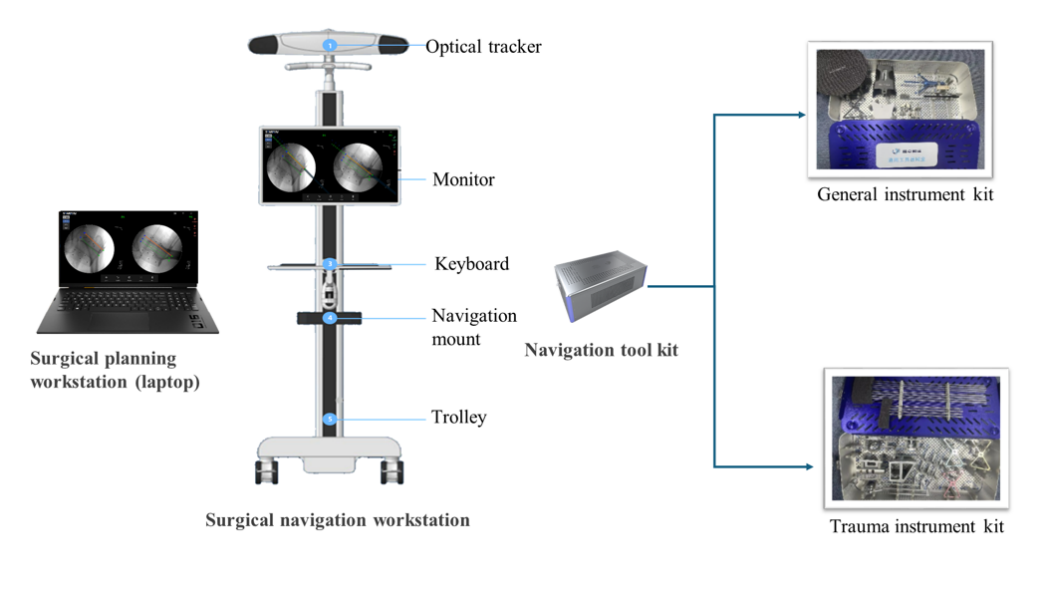
Main equipment of the navigation system.

The navigation system primarily consists of a surgical planning workstation (laptop computer), a surgical navigation console, and a navigation instrument table. The entire setup has a minimal footprint and features user-friendly operation. The main procedural workflow proceeds as follows. First, anteroposterior and lateral views of the target lesion area are acquired, ensuring that all marker spheres of the image registration device and the target lesion area are included. Next, after determining whether distortion correction is needed based on the intraoperative use of a C-arm (equipped with a flat-panel detector or image intensifier), registration verification and trajectory planning for TCS are performed on the workstation system. This system enables simultaneous planning of all TCS, eliminating the need for repeated adjustments to optimize their spatial relationship and thereby reducing surgical time. Finally, guide wires are implanted along the planned trajectories, and subsequently, depth measurement and TCS insertion are completed (Figure 2).

It must be emphasized that although the novel navigation system can provide visual assistance for fracture reduction, manual reduction will be exclusively used in this study to minimize variability and potential bias, thereby safeguarding study quality. The navigation system will be primarily used for surgical planning and screw placement.

### Sample Size

The sample size was calculated using the following equations:




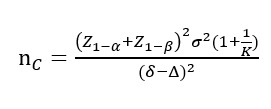


**(1)**




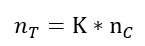


**(2)**

In these equations, 1–α denotes the significance level, *1–β* represents the statistical power, *Z_1−α_* and *Z_1−β_* are critical values derived from the Z-table, *σ* refers to the SD of the 2 groups, *δ* indicates the mean difference between groups, and *Δ* stands for the superiority margin.

According to published literature, the mean number of intraoperative fluoroscopies in screw fixation for FNFs is approximately 32.2 times [26], while the use of a navigation system is expected to significantly reduce fluoroscopy frequency. On the basis of preliminary pilot data and the experience with navigated procedures at our institution, the mean (SD) number of fluoroscopy images for the control group (nonnavigated) is 32.2 (SD 5.2), and that for the experimental group (navigated) is 15.2 (SD 3.7). The superiority margin was set at 15.4, with a 1-sided significance level of .025 and a statistical power of 0.8.

With a 1:1 allocation ratio between the 2 groups, the sample size calculation was performed using the aforementioned formula and PASS (version 15.0; NCSS LLC) software. Considering patient recruitment feasibility, each group required 126 participants, yielding a total of 252 subjects. After accounting for a 15% dropout rate and distribution across multiple centers, the final sample size was set at 150 (50%) per group, resulting in a total of 300 participants.

### Allocation

This multicenter trial will adopt a competitive enrollment model conducted concurrently at all participating sites. Initial participant distribution will strive for relative balance to ensure adequate representation across centers. As the study progresses, site-specific enrollment targets may be revised to maintain intercenter equilibrium, accounting for practical constraints and variations in recruitment rates. Centers recruiting fewer than 10 participants in either the control or experimental arm will be analytically grouped according to China’s geographic and demographic patterns. Sites contributing more than 50% of the total sample will be subject to investigator-stratified subgroup analyses.

### Randomization and Blinding

This is a single-blind trial in which patients and outcome assessors will be blinded to treatment allocation, while operating surgeons cannot be blinded because of the distinct intraoperative workflows associated with the navigation system and the freehand technique. Patients will remain blinded to their group assignment throughout the surgical procedure and the entire 12-month follow-up period, with the specific technique disclosed only after completion of all outcome assessments unless early unblinding is required for safety reasons. Radiographic assessors and functional outcome assessors will be blinded to group allocation, as all imaging studies and clinical assessments will be coded with unique participant identifiers and conducted without access to treatment assignment records. The primary statistical analyst will remain blinded until the final database is locked and all predefined analyses are completed, with group codes revealed only after the statistical analysis plan has been fully executed. Emergency unblinding may be performed by the principal investigator in the event of a serious adverse event for which knowledge of the intervention is essential for patient management, and any such event will be documented and reported.

Randomization will be performed by the coordinating center using a centralized computer-generated system with permuted blocks of 4 to ensure balanced allocation across treatment groups. Assignment details will be distributed to all participating sites via sequentially numbered, sealed opaque envelopes. All surgical procedures will be performed in accordance with the assigned randomization.

### Recruitment Strategy and Patient Screening

Before the study begins, baseline characteristics and medical history will be recorded for all potential participants. Those who meet the eligibility criteria (Textbox 1) will receive detailed study information, including the study purpose, procedures, risks, and benefits. Informed consent will then be obtained voluntarily. Eligible subjects who meet all criteria will be formally enrolled. They will then be assigned to treatment groups according to the site’s randomization scheme. After entering the electronic randomization system, all subject data will be locked. Each operation within the system will be automatically recorded in audit trails for monitoring purposes (Figure 1).

**Eligibility criteria for the study.**

**Inclusion criteria**

- Aged ≥18 years, regardless of gender
- Diagnosed with a femoral neck fracture
- Indicated for and medically fit to undergo internal fixation surgery
- Provided written informed consent, either by the patient or their legally authorized representative

**Exclusion criteria**

- Prior participation in another clinical trial within the period prior to the primary end point assessment
- Known allergy or hypersensitivity to ≥1 components of the implant materials
- Deemed by the investigator to be medically unfit or too frail to tolerate the surgical procedure due to systemic comorbidities
- Presence of active infective foci in the hip or other parts of the body, as determined by the investigator
- Diagnosis of metabolic bone disease, radiation-induced bone disease, or similar conditions
- Severe hip contracture or significant muscle weakness with long-standing, painless fusion in a functional position
- Presence of inflammatory arthritis, such as rheumatoid arthritis, systemic lupus erythematosus–associated arthritis, or ankylosing spondylitis
- Cognitive impairment, inability to comprehend study requirements, or anticipated poor compliance
- Any other condition that, in the judgment of the investigator, renders the patient unsuitable for study participation

### Trial Intervention Procedures

Eligible participants will be randomized to receive either TCS fixation or NS+TCS fixation.

#### Control Group: TCS Fixation

Following the induction of anesthesia, the patient is placed in a supine position on a traction table. Under fluoroscopic guidance with a G-arm, traction reduction of the FNF is performed until satisfactory reduction is achieved. The surgical site is routinely disinfected and draped in sterile sheets.

A lateral hip approach is made through 3 separate incisions, each approximately 1 cm in length. The skin and subcutaneous tissues are incised sequentially, followed by incision of the tensor fasciae latae and blunt dissection of the muscle. Under fluoroscopic guidance, 3 Kirschner wires are inserted from the lateral aspect of the left thigh, through the FNF site starting above the level of the lesser trochanter, and advanced into the subchondral bone of the femoral head in an inverted triangular configuration. After depth measurement, TCSs are inserted over the guide wires to fix the fracture. Postoperative fluoroscopy is used to confirm satisfactory fracture reduction and alignment, as well as appropriate positioning of the internal fixation screws. The Kirschner wires are then removed. The incision is irrigated thoroughly, and hemostasis is confirmed. After ensuring that the count of surgical instruments and gauze is correct, the incision is closed in layers.

#### Intervention Group: NS+TCS Fixation

Following successful anesthesia, the patient is positioned supine on a traction table. Satisfactory reduction of the fracture fragments is achieved under G-arm fluoroscopic guidance. The operative field is routinely prepared and draped in a sterile fashion.

Two guide pins are inserted, one at the anterior superior iliac spine and another at the lateral femoral condyle, to serve as anchoring points. A navigation reference array is then mounted. The exposed ends of the guide pins are trimmed to an appropriate length to avoid interference with subsequent fluoroscopic imaging. Image registration markers are positioned anteriorly and laterally to the hip joint. Standard anteroposterior and lateral hip radiographs are acquired and uploaded to the navigation system workstation for preoperative trajectory planning for TCS placement. An inverted triangular configuration is selected for dispersed screw placement. After calibration of the navigated drill sleeve, the planned trajectories are used to percutaneously insert 3 guide wires sequentially into the femoral neck and head through small skin incisions. Subsequently, TCS are inserted over the guide wires. Fluoroscopic confirmation is obtained to verify satisfactory screw length and position.

**Figure 3:**
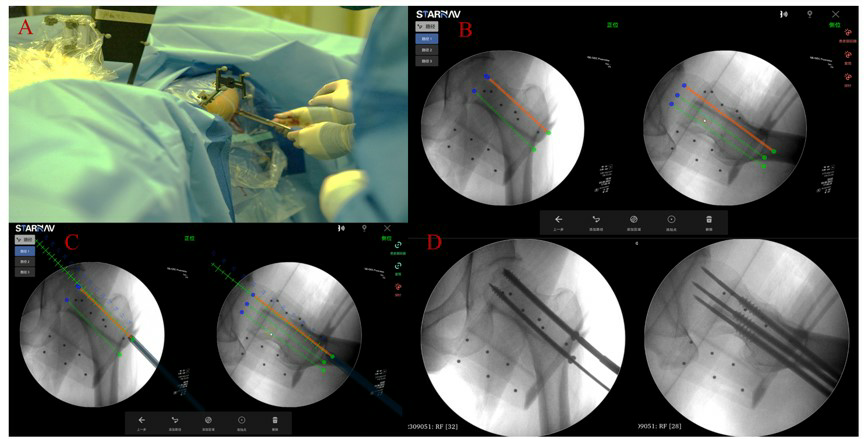
Core navigational procedure: (A) placement of the reference array and creation of the surgical incision; (B) planning of screw trajectory, including position, angulation, and orientation; (C) guided K-wire insertion with concurrent depth measurement; and (D) screw implantation.

Upon confirmation of adequate fixation, the reference guide pins at the anterior superior iliac spine and lateral femoral condyle are removed. The surgical incisions are irrigated and closed in layers (Figure 3).

### Postoperative Management Protocol

Postoperative full weight-bearing may be restricted during the initial 2 weeks for both limbs. Ambulation aids, such as canes or walkers, will be provided according to individual functional needs. Standard deep vein thrombosis prophylaxis will be routinely administered, accompanied by a 3-day perioperative antibiotic regimen to prevent surgical site infection.

### Outcomes and Measures

#### Primary Outcome

The total number of intraoperative fluoroscopic images will be recorded for both groups. This includes each anteroposterior and lateral view obtained, starting from fracture reduction. To minimize observer bias, fluoroscopic counts will be categorized and analyzed separately for fracture reduction, navigational positioning, and implant placement.

Fluoroscopy frequency was selected as the primary outcome for this superiority trial for the following reasons. First, the number of intraoperative fluoroscopic images is an objective, quantifiable, and operator-independent measure that directly reflects the efficiency and precision of guide wire and screw placement. Second, reducing radiation exposure is a major clinical advantage of navigation-assisted surgery, as it lowers occupational radiation risk for surgical staff and potential patient harm. Finally, fluoroscopy frequency is significantly correlated with operative time, surgical accuracy, and the learning curve, making it a valid surrogate end point for overall surgical quality. Therefore, a reduction in fluoroscopy frequency with the navigation system, compared with the freehand technique, would support the superiority of the navigation system in terms of surgical efficiency and safety.

#### Secondary Outcomes

The secondary outcomes are as follows:

- Neck-shaft angle: defined as the angle between the longitudinal axis of the femoral shaft and the axis of the femoral neck, as measured on postoperative radiographs
- Guide wire placement quality (TCS groups): classified according to guide wire arrangement as 3 parallel, 2 parallel, or nonparallel, as determined from intraoperative imaging
- Operative time: measured from the initial skin incision to complete wound closure, as documented in intraoperative records
- Intraoperative blood loss: quantified as the total blood volume collected by the suction apparatus during the operation
- Fracture healing status: assessed using anteroposterior and lateral hip radiographs at 1, 3, 6, and 12 months and computed tomography scans at 1 and 3 months by evaluating implant stability, disappearance of the fracture line, and absence of displacement at the fracture site
- Zarit Caregiver Burden Score: evaluated using an established questionnaire to assess the perceived level of caregiving burden experienced by caregivers of patients after a fracture
- Harris Hip Score: assessed using the Harris scoring system during outpatient visits at 1, 3, 6, and 12 months postoperatively to evaluate hip joint function
- 36-Item Short Form Health Survey Score: assessed using the 36-Item Short Form Health Survey at 1, 3, 6, and 12 months postoperatively to reflect the patient’s overall physical and mental health trajectory

#### Safety Events

The safety end points mainly include the incidence of device-related adverse events, device deficiencies, adverse events, and serious adverse events. These outcomes will be evaluated over the entire follow-up period and are defined as follows:

- Device-related adverse event: a problem caused by the test medical device
- Device deficiency: any issue with the investigational device that could pose an unacceptable risk to patient health or safety during normal use, including deficiencies in labeling, manufacturing, malfunction, or performance failure
- Adverse event: any negative health change occurring in participants during this study, regardless of whether it is linked to the test device
- Serious adverse event: any adverse event resulting in death, a life-threatening situation, persistent, significant disability or incapacity, requiring hospitalization or extend existing hospitalization, or requiring medical or surgical intervention to prevent permanent damage

All outcomes measures are listed in Table 1.

**Table 1:** Summary of all outcome measures.

Outcome categories and measures			Definition		Assessment time points
Primary outcomes: fluoroscopy frequency			Total number of intraoperative fluoroscopic images acquired during the procedure		Intraoperative period
**Secondary outcomes**					
	Neck-shaft angle	Angle between the femoral shaft axis and the femoral neck axis		Intraoperative period	
	Guide wire placement accuracy	Classification of guide wire arrangement as 3 parallel, 2 parallel, or nonparallel		Intraoperative period	
	Surgical duration	Time from skin incision to wound closure		Intraoperative period	
	Intraoperative blood loss	Total blood loss collected through suction		Intraoperative period	
	Fracture healing status	Fracture healing assessed by implant stability, disappearance of the fracture line, and absence of displacement		1, 3, 6, and 12 months	
	Zarit Caregiver Burden Score	Assessment of caregiver burden		1, 3, 6, and 12 months	
	Harris Hip Score	Assessment of hip function recovery		1, 3, 6, and 12 months	
	36-Item Short Form Health Survey Score	Assessment of health-related quality of life		1, 3, 6, and 12 months	
**Safety events**					
	Device-related adverse events	Adverse events directly related to the study device		All follow-ups	
	Serious adverse events	Events causing death, disability, or hospitalization		All follow-ups	
	Device defects	Malfunction or labeling error that may pose a health risk		Intraoperative and follow-up	
	General adverse events	Any unfavorable medical event regardless of causality		All follow-ups	

### Follow-Up Visits

During the postoperative follow-up period, patients will be evaluated through scheduled outpatient visits. Wound healing will be assessed at 2 weeks after surgery. Radiographic examinations will be performed at 1, 3, 6, and 12 months postoperatively, while computed tomography scans will be obtained at 6 and 12 months after surgery. These imaging studies are reviewed to determine the position of the internal fixation system and the status of fracture healing. Functional scoring of the affected limb and general health assessments will also be conducted during these visits. The complete follow-up schedule is presented in Table 2.

**Table 2:** Study procedures and visiting schedule.

Study activities		Screening (day −7 to 0)	Surgery (day 0)	Postoperative follow-ups				
				2 weeks	1 month+2 weeks or −2 weeks	3 months+2 weeks or −2 weeks	6 months+2 weeks or −2 weeks	12 months+2 weeks or −2 weeks
**Enrollment and consent**								
	Informed consent	✓						
	Demographic information	✓						
	Vital signs	✓	✓	✓				
	Medical history	✓						
	Laboratory tests	✓						
	Garden classification	✓						
	Time from injury to surgery	✓	✓					
**Surgical details**								
	Intraoperative blood loss		✓					
	Duration of surgery		✓					
	Guide pin implantation effect		✓					
	Intraoperative fluoroscopy (3-pin)		✓					
	Intraoperative fluoroscopy (total)		✓					
	Neck-shaft angle		✓					
	Reduction quality (satisfaction)		✓					
	Garden alignment index		✓					
	Implant handling evaluation		✓					
	Surgery-related record		✓					
**Clinical and imaging assessments**								
	Radiography	✓		✓	✓	✓	✓	✓
	Computed tomography scan	✓		✓			✓	✓
	Harris Hip Score	✓		✓	✓	✓	✓	✓
	36-Item Short Form Health Survey quality-of-life score	✓		✓	✓	✓	✓	✓
	Zarit Caregiver Burden Scale	✓		✓	✓	✓	✓	✓
	Local physical examination	✓		✓	✓	✓	✓	✓
	Rejection reaction		✓	✓	✓	✓	✓	✓
	Fracture healing				✓	✓	✓	✓
	Weight-bearing status				✓	✓	✓	✓
	Femoral neck shortening			✓			✓	✓
	Wound healing assessment			✓	✓			
**Safety monitoring**								
	General adverse events	✓	✓	✓	✓	✓	✓	✓
	Serious adverse events	✓	✓	✓	✓	✓	✓	✓
	Device-related issues		✓					
	Concomitant medications or treatments	✓	✓	✓				

### Data Collection

From preoperative screening to the end of follow-up, all patient information will be collected using standardized case report forms. These data will be entered into the National Real-World Research Platform for Orthopedics, Sports Medicine, and Rehabilitation. To support quality assurance and follow-up tracking, user accounts on the platform will have differentiated access privileges. Data managers will review all entries in the case report forms. If any inconsistencies are identified, queries will be generated and sent to the investigator through the clinical research associate using standardized data query forms. The data manager will make database changes or confirmations based on the investigator’s feedback. If more details are needed, further queries may be issued.

### Data Integrity

After data entry and validation, the data manager, principal investigator, project team, and statisticians will review the final database. This team will verify the database’s accuracy and integrity. Their review will result in the final definition of the analysis populations. The platform also includes features for follow-up reminders and oversight.

### Early Withdrawal

Participants may be withdrawn from the study for any of the following reasons: (1) incorrect enrollment of patients failing to meet inclusion or exclusion criteria, (2) nonadherence to required study procedures among eligible patients after enrollment, and (3) protocol deviations or incomplete data that compromise efficacy or safety assessments.

### Statistical Plan

All statistical analyses will be performed using SPSS (version 26.0; IBM Corp), R (version 4.2.0; R Foundation for Statistical Computing), and Origin 2021 (version Origin 2021; OriginLab Corp), with a 2-sided significance level of *P*<.05. For the primary outcome (fluoroscopy frequency), group comparisons will be performed using a 1-sided *t* test or a Mann-Whitney *U* test, supplemented by a linear mixed-effects model with study site as a random effect to account for center-level clustering. Continuous secondary outcomes will be analyzed using similar linear mixed-effects models with baseline covariates, while categorical outcomes will be analyzed using generalized linear mixed-effects models with study site as a random intercept. For outcomes assessed repeatedly at 1, 3, 6, and 12 months, longitudinal mixed-effects models will include fixed effects for treatment group, time, and the group-by-time interaction, along with subject-specific random intercepts and slopes nested within site. Any baseline variable with a standardized difference >0.10 will be adjusted as a covariate. The center-by-treatment interaction will be formally tested; if significant (*P*<.10), exploratory subgroup analyses stratified by site will be conducted. Predefined subgroup analyses include age, gender, Garden classification, and fracture side. Sensitivity analyses will include per-protocol and as-treated analyses.

### Ethical Considerations

#### Ethics Committee Approval

This study received initial ethics approval from the institutional review board of the coordinating center, the Chinese People’s Liberation Army General Hospital (S2024-341-01, version V1.0, dated May 1, 2024). This multicenter randomized controlled trial will be carried out only after each participating site obtains separate approval from its local ethics committee. No study procedures will begin at any site prior to written confirmation of ethics approval. The study has been prospectively registered on ClinicalTrials.gov (NCT06713018).

#### Informed Consent

Written informed consent will be obtained from every participant or a legally authorized representative before any study-related procedures are performed. All eligible patients will receive comprehensive information regarding the study purpose, procedures, potential risks and benefits, alternative treatments, and the voluntary nature of participation. Patients will be explicitly informed that refusal to participate or withdrawal at any time will not affect their subsequent medical care. Consent forms will be signed and dated by both the participant and the investigator obtaining consent. For patients with cognitive impairment who are unable to provide consent but meet the inclusion criteria, consent will be obtained from a legally authorized representative in accordance with local regulations.

#### Privacy and Confidentiality

All patient data collected during this trial will be deidentified using unique participant codes, with a separate password-protected linkage file maintained by the principal investigator at each site. Paper records will be stored in locked cabinets, and electronic data will be entered into a secure platform (National Real-World Research Platform for Orthopedics, Sports Medicine, and Rehabilitation) with role-based access control and audit trails. No individual participant data that could allow reidentification will be publicly disclosed.

#### Participant Compensation

Participants may receive direct medical benefits, including early rehabilitation guidance and priority follow-up. The navigation system is provided free of charge by the Visual MedTech Co Ltd. Routine clinical costs will be charged according to national regulations. Patients completing the full study will receive a one-time stipend of RMB 400 (RMB 1=US $0.15 as of June 15, 2026) after the final follow-up, which will be covered by the funding project. Cost associated with device-related adverse events will be covered by the sponsor according to the study’s insurance arrangements.

### Dissemination

All proprietary information and materials provided by the research project team, including manufacturing processes, foundational scientific data, medical device specifications, and unpublished findings, will be treated as strictly confidential. All individuals and entities involved in the study are subject to confidentiality obligations. Disclosure to third parties is expressly prohibited without prior written authorization from the project team. These obligations remain binding following study termination or completion. No patient or public representatives participated in the design, conduct, reporting, or dissemination of this research.

## Results

This multicenter, prospective, randomized controlled trial was initiated in June 2024. Enrollment is scheduled to be completed by October 2026, with a target sample size of 300 participants. The final 12-month follow-up is expected to be completed by October 2027. Data cleaning and database locking will be performed within 3 months thereafter, followed by analyses of the primary and secondary outcomes.

The feasibility of implementing this protocol in future larger-scale studies will be evaluated using the following metrics: (1) navigation system performance will be assessed for each procedure in the intervention group, including trajectory planning accuracy, reference array stability, first-pass guide wire success rate, and the incidence of technical failures or device deficiencies; (2) retention rates will be assessed through completion of scheduled follow-up visits, imaging protocols, and patient-reported outcome measures; and (3) the volume of intraoperative data collected—including fluoroscopy frequency, operative time, blood loss, neck-shaft angle, and guide wire placement quality—will be quantified to inform data management strategies in future investigations.

Any deviations from the approved protocol will be documented and categorized as major or minor. All deviations will be reported to the coordinating center’s institutional review board and to the ethics committees of participating sites. Moreover, the clinical value of incorporating the navigation system will be determined by comparing the primary and secondary outcomes between the 2 treatment groups after trial completion. The results of this trial will provide evidence on the viability of navigation-assisted surgery using a fluoroscopy-based, workflow-preserving system for FNF fixation.

Upon completion of the trial, the results will be submitted for publication in peer-reviewed orthopedic or surgical journals, regardless of the direction of the outcomes. The coordinating center will be responsible for drafting the primary manuscript. There are no intended restrictions on the publication of results. This trial protocol has been prospectively registered on ClinicalTrials.gov (NCT06713018), and any significant protocol amendments will be documented and publicly disclosed.

## Discussion

### Principal Findings

This multicenter, prospective, randomized controlled trial is designed to determine whether the StarNav-1 intelligent navigation system is superior to the conventional freehand technique for TCS fixation of FNF. Upon completion, we anticipate that the primary outcome will be significantly lower in the navigation-assisted group than in the freehand group. This hypothesis is informed by prior studies that have reported reductions in radiation exposure with navigation-assisted techniques [15,21,27,28]. On the basis of the existing literature, we also anticipate that secondary outcomes, including guide wire placement quality, neck-shaft angle restoration, operative time, and intraoperative blood loss, will favor the navigation group [16-18]. However, as this is a protocol for an ongoing trial, all findings remain to be empirically tested.

### Comparison to Prior Work

The anticipated findings of this trial would align with a growing body of evidence supporting navigation-assisted fixation of FNF. Gao et al [22] reported that a laser-guiding navigation system significantly reduced radiation exposure duration (35 s vs 110 s in the freehand group; *P*<.001) and shortened operative time (40-45 min vs 65-75 min; *P*=.02) [15]. Recent studies of other navigation systems have reported primary success rates ranging from approximately 93% to 99% [16]. In terms of accuracy, Schep et al [19] reported that a fluoroscopy-based navigation system achieved an accuracy of 1.17 mm, while He et al [18] reported angular errors as low as 1.08° in the coronal plane and 1.25° in the axial plane using a biplanar robotic navigation system.

The novel StarNav-1 system differs from prior navigation technologies in several aspects. Unlike optical navigation systems that rely on optical tracking and image registration, which require specialized equipment, prolonged registration times, elevated costs, and therefore have experienced limited widespread adoption, the StarNav-1 is designed to preserve the conventional surgical workflow and uses only intraoperative fluoroscopy. This distinction addresses a key gap identified in the literature, as current techniques still fall short of simultaneously meeting the requirements for procedural simplicity, high accuracy, and minimal radiation exposure..

### Strengths and Limitations

This trial has several methodological strengths. The multicenter design enhances generalizability across different hospital settings. Prospective randomization with concealed assignment minimizes selection bias. Blinded outcome assessment for patients, radiographic reviewers, and functional assessors reduces detection bias. The sample size calculation was based on published literature [27] and a prespecified superiority margin, ensuring adequate power for the primary outcome.

Several limitations should be acknowledged. First, operating surgeons cannot be blinded because of the distinct intraoperative workflows associated with the navigation system and the freehand technique, which may introduce performance bias. However, the primary outcome is an objective, quantifiable measure that minimizes the impact of this bias. Second, the 12-month follow-up period may be insufficient to detect very late complications, such as osteonecrosis of the femoral head, which can occur beyond 1 year. Finally, all participating centers are in China, and findings may not be directly generalizable to health care systems with different surgical training practices or equipment.

### Future Directions

Depending on the results of this trial, several future research directions are anticipated. If the superiority hypothesis is confirmed, a cost-effectiveness analysis will be warranted to determine whether the clinical benefits justify the additional cost of the navigation system. A cluster randomized trial or a stepped-wedge design could evaluate the impact of implementing the system at the institutional level. Subgroup analyses may identify patient populations that derive the greatest benefit. Furthermore, if the system performs well for FNF, its application to other indications could be explored in future trials. Finally, integration of artificial intelligence for automated trajectory planning or augmented reality visualization represents potential next-generation enhancements to the current fluoroscopy-based navigation platform.

## Abbreviations

FNF: femoral neck fracture
NS+TCS: navigation system–assisted three cannulated screws
TCS: three cannulated screws

## Data Availability

The datasets generated and/or analyzed during this study will not be publicly available due to the ongoing trial and patient confidentiality protections. However, deidentified individual participant data and a data dictionary defining each field in the dataset will be made available upon reasonable request to the corresponding author, following approval by the institutional review board of the Chinese People’s Liberation Army General Hospital and execution of a data-sharing agreement. Data will be shared for noncommercial, academic purposes only. No publicly accessible repository or code sharing is applicable at this protocol stage.
